# Editorial: Biomarkers of neurodegeneration and brain function and their relationships with clinical and neuropsychological outcomes in monitoring deep brain stimulation efficacy in movement disorder patients

**DOI:** 10.3389/fneur.2022.936706

**Published:** 2022-08-03

**Authors:** Javier J. González-Rosa, Francisco Escamilla-Sevilla, Letizia Leocani

**Affiliations:** ^1^Psychophysiology and Neuroimaging Group, Institute of Biomedical Research Cádiz (INiBICA), Cádiz, Spain; ^2^Department of Psychology, University of Cadiz, Cadiz, Spain; ^3^Movement Disorders Unit, Department of Neurology, Virgen de las Nieves University Hospital, Institute of Bio-Health Research (ibs.Granada), Granada, Spain; ^4^University Vita-Salute San Raffaele and Experimental Neurophysiology Unit, Institute of Experimental Neurology-INSPE, Scientific Institute San Raffaele, Milan, Italy

**Keywords:** deep brain stimulation, Parkinson's disease, movement disorders, local field potentials, subthalamic nucleus (STN), neuroimaging, biomarkers

Deep brain stimulation (DBS) is a widely used therapy for a variety of movement disorders, such as Parkinson's disease (PD), Essential Tremor (ET), and dystonia, mainly targeting the subthalamic nucleus (STN), thalamic ventral intermediate nucleus (Vim), and globus pallidus internus (Gpi). Although this treatment has been available for decades, studies on early- or long-term patient outcomes have been still limited. While DBS has evolved into an evidence-based standard treatment for movement disorders, the identification of preoperative or postoperative predicting factors in DBS candidates would be of imperative clinical value, allowing us to monitor surgical performance and improve surgical outcomes. Because of several gaps in understanding DBS mechanisms, measuring its effects and efficacy is still of great interest. Therefore, the use of electrophysiological, neuroimaging, and molecular investigations could be useful to provide biomarkers to determine the effects of DBS on patients with movement disorders.

Taking this into consideration, the Research Topic “*Biomarkers of neurodegeneration and brain function and their relationships with clinical and neuropsychological outcomes in monitoring deep brain stimulation efficacy in movement disorder patients*” by Frontiers in Neurology contributes to the debate by providing updates and a variety of viewpoints on this important theme, which was explored throughout seven articles. These updates focus on exploring reliable and potential markers and prediction indexes for DBS outcomes of movement disorder patients from multidisciplinary perspectives including neuroimaging techniques and invasive electrophysiological recordings.

Although the clinical features and the wide collection of signs and symptoms of the main movement disorders conditions are well-known, there is still much debate over whether an etiological link between different movement neurological diseases exists and whether they share a common pathophysiology. Huang et al. underlined the importance of serum brain-derived neurotrophic factor (BDNF) levels—a marker for neuroprotection and neuroregeneration in adulthood—and its involvement in the pathophysiology and severity of restless legs syndrome (RLS) in PD. This study showed that decreased serum BDNF levels may be involved in the pathophysiology of RLS in PD patients, which advocates identifying accurate biomarkers, including fluid biomarkers in neurodegeneration, for providing better diagnostic value, clinical intervention, and disease monitoring during pharmacological and stimulation therapies. Wang et al. focused on freezing of gait (FOG) symptoms in PD by quantifying the changes in resting-state functional connectivity through magnetic resonance imaging (MRI). They delved into the finding that dynamic functional connectivity between the thalamic nuclei and relevant associative cortical areas involved in sensorimotor integration and cognitive function was disrupted in PD-FOG. Importantly, altered dynamic function between the left intralaminar nuclei and the right inferior parietal lobule and between the left medial geniculate and left postcentral gyrus was associated with the development of FOG in PD patients, a finding that could have further implications in the design of therapeutical strategies and to refine DBS targeting. He et al. detailed two cases in which bilateral subthalamic stimulation was tested in relation to Pisa syndrome, a major postural abnormality in PD patients. Despite the lack of understanding of the pathophysiological mechanism of underlying Pisa syndrome, an almost complete resolution of tremor and rigidity symptoms as well as of lateral trunk flexions was achieved with STN DBS, highlighting its potential as a treatment for postural abnormalities in PD. Geraedts et al. explored the clinical effects associated with DBS therapy in patients diagnosed with PD, dystonia, tremor, or a combination of these disorders by comparing the thresholds for inducing side-effects between intraoperative test stimulation and postoperative chronic stimulation during awake Vim DBS and in GPi DBS. This large-scale retrospective investigation demonstrated that, despite individual heterogeneity, there are no differences in the induction of both capsular and non-capsular side effects between the two assessments, for both patients of GPi and Vim DBS.

Anatomically, the human STN has been traditionally divided into sensorimotor, associative, and limbic subterritories, which may explain the wide range of clinical effects of DBS as well as the diversity of physiological tasks in which the STN has been identified to be engaged. Wei et al. compared abnormal neuronal activity collected intraoperatively from local field potential (LFP) recordings in both dorsomedial and dorsolateral STN, demonstrating that the specific features of gamma LFP activity may be used to differentiate STN subterritories, which could contribute to better understanding the effects of dorsomedial STN DBS for PD. These findings might help in clinical decision-making during the intraoperative selection of the optimal lead location and perhaps speed up the adjustment of postoperative DBS settings. Alonso-Frech et al. similarly investigated the use of DBS and neuroimaging techniques for those proportion of PD patients who suffer transient or permanent neurobehavioral adverse effects after subthalamic stimulation. This study examined the effects of directional DBS in a patient case report of someone who exhibited excessive mirthful laughing during left ring STN stimulation, which ceased when switched to directional stimulation. The methodological approach of this study, combining biomarkers of LFP beta oscillations from implanted macroelectrodes in conjunction with tractography findings, might offer a distinctive chance to guide neurostimulation toward more convenient motor areas, minimizing non-motor side effects. Sanmartino et al. expanded our knowledge about the link between STN beta activity, a well-established physiomarker of PD severity, with regional gray matter volume along with cortical thickness. The findings of this study provide support to further research into how increased synchronization at beta frequencies within the STN may be associated with both GM volume decreases in dorsal striatal motor circuits, principally the right putamen, and more widespread thinning of prefrontal associative areas. Moreover, these results emphasize the notion that neurodegeneration in the cortical-basal ganglia loop, particularly dorsal striatum gray matter volume and prefrontal cortical thickness, may be related to PD progression, which could be used as an MRI-based marker of disease progression and the effect of treatments.

Taken together, these studies updated new advances about DBS biomarkers and treatment response using a variety of methods (blood proteins, electrophysiology, and neuroimaging) in PD and other movement clinical conditions to identify alterations with specific traits of the disease or with the outcome of the DBS efficacy, expanding the number of biomarkers to consider in the optimization of deep brain surgery in movement disorders.

## Author contributions

All authors conceived and developed the presented ideas and contributed to the final manuscript equally.

## Funding

This work was supported by the Spanish Ministry of Science, Innovation and Universities (MICINN) under Grants (RYC-2015-18467 and PSI2017-85951-R) and by the European Regional Development Fund through the Andalusian Ministry of Health and Families under Grants (PI-0025-2017 and PI-0034-2019).

## Conflict of interest

The authors declare that the research was conducted in the absence of any commercial or financial relationships that could be construed as a potential conflict of interest.

## Publisher's note

All claims expressed in this article are solely those of the authors and do not necessarily represent those of their affiliated organizations, or those of the publisher, the editors and the reviewers. Any product that may be evaluated in this article, or claim that may be made by its manufacturer, is not guaranteed or endorsed by the publisher.

